# A244 DEVELOPING AND ASSESSING THE EFFECTIVENESS OF A REMOTE MONITORING PROTOCOL FOR ULCERATIVE COLITIS PATIENTS - ULCERATIVE COLITIS CLINICAL OUTREACH (UCCO)

**DOI:** 10.1093/jcag/gwad061.244

**Published:** 2024-02-14

**Authors:** S A MacKay, C Hagerman, D Parsons, L Dieleman, F Hoentjen, K Kroeker, F Peerani, K Wong, B Halloran

**Affiliations:** University of Alberta Division of General Internal Medicine, Edmonton, AB, Canada; University of Alberta Department of Emergency Medicine, Edmonton, AB, Canada; University of Alberta Division of Gastroenterology, Edmonton, AB, Canada; University of Alberta Division of Gastroenterology, Edmonton, AB, Canada; University of Alberta Division of Gastroenterology, Edmonton, AB, Canada; University of Alberta Division of Gastroenterology, Edmonton, AB, Canada; University of Alberta Division of Gastroenterology, Edmonton, AB, Canada; University of Alberta Division of Gastroenterology, Edmonton, AB, Canada; University of Alberta Division of Gastroenterology, Edmonton, AB, Canada

## Abstract

**Background:**

Ulcerative colitis (UC) is a chronic inflammatory bowel disease which requires regular gastroenterologist monitoring. Outpatient monitoring programs to date have focused on clinical scores alone, leaving patients with asymptomatic inflammation susceptible to undertreatment despite increased risk of flares and colorectal cancer. The University of Alberta IBD Unit developed and piloted an outreach and remote monitoring protocol for UC patients including clinical and biochemical variables. We assessed the effectiveness of this protocol at improving UC care.

**Aims:**

This study aimed to develop a clinical outreach and remote patient monitoring protocol for UC patients and assess the impact this protocol had on disease management.

**Methods:**

Biologic naïve adult UC patients who had not been reviewed by their gastroenterologist in at least six months were contacted by phone and mailed monitoring kits with Partial Mayo, Sutherland Index, and medication adherence questionnaires and a requisition for blood work and fecal calprotectin (FCP). Results were compiled and sent to each patient’s gastroenterologist. Participating gastroenterologists completed a survey about the perceived utility of the protocol and intended UC management changes.

**Results:**

85 patients completed the protocol. 86.4% of physician surveys rated the protocol as helpful to clinicians. UC management was changed in 55.6% of cases, with 82.2% of changes being management escalations. Six patients had active flares and 17 patients with asymptomatic inflammation were identified. Endoscopy was completed for 23 patients, with active disease observed in 78.2% of cases. Six patients started biologic therapies based on the protocol and endoscopic findings. UC management escalations were significantly predicted by FCP and Sutherland Index scores on logistic regression analysis.

**Conclusions:**

This protocol had a meaningful impact on UC management and identified patients with active disease in both the presence and absence of clinical remission. Remote outpatient monitoring in UC should include collection of both FCP and clinical scores every six months to improve UC management.

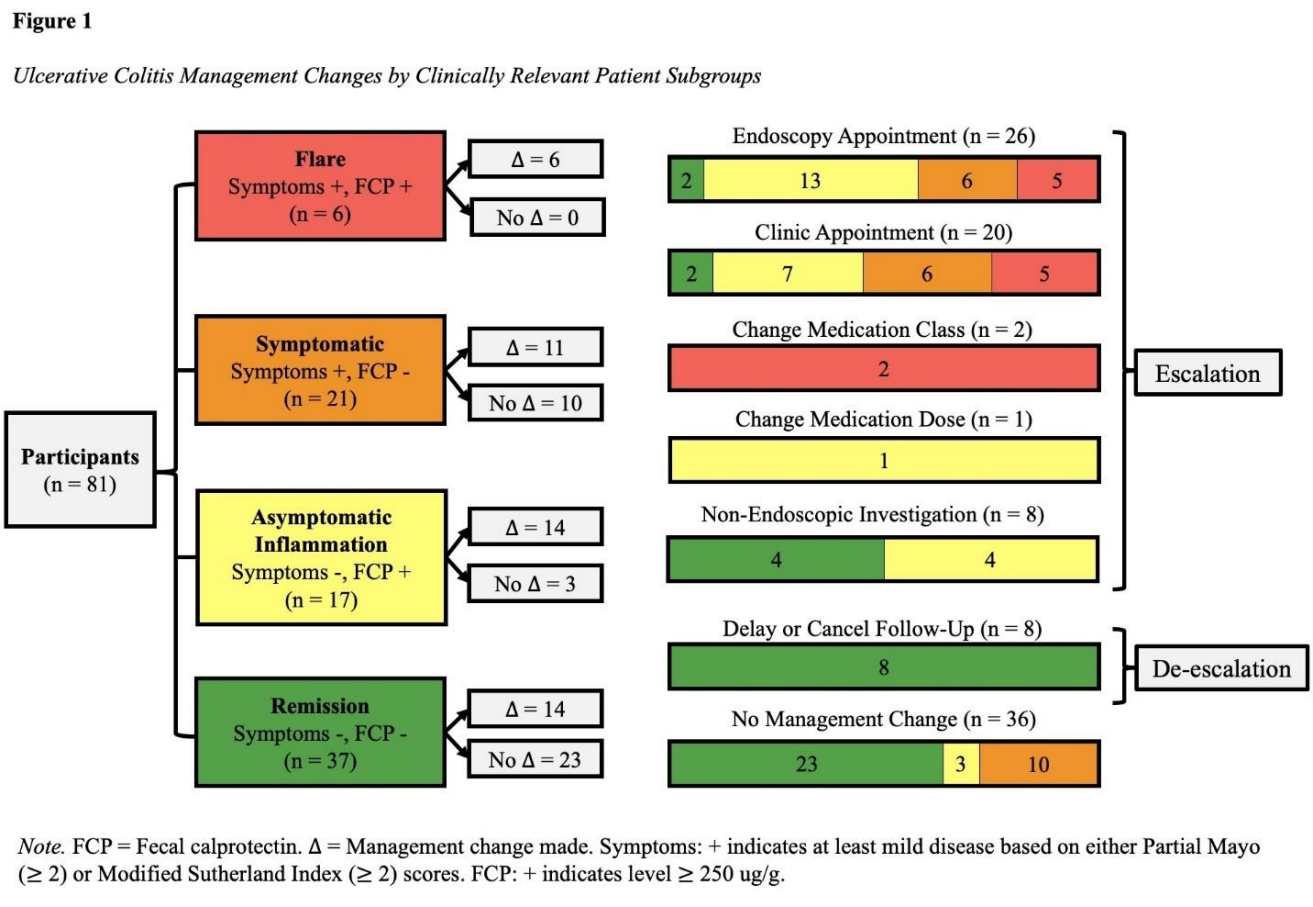

**Funding Agencies:**

None

